# Polymicrobial Ventilator-Associated Pneumonia: Fighting *In Vitro Candida albicans-Pseudomonas aeruginosa* Biofilms with Antifungal-Antibacterial Combination Therapy

**DOI:** 10.1371/journal.pone.0170433

**Published:** 2017-01-23

**Authors:** Maria E. Rodrigues, Susana P. Lopes, Cláudia R. Pereira, Nuno F. Azevedo, Anália Lourenço, Mariana Henriques, Maria O. Pereira

**Affiliations:** 1 Centre of Biological Engineering, LIBRO–Laboratório de Investigação em Biofilmes Rosário Oliveira, University of Minho, Campus de Gualtar, Braga, Portugal; 2 LEPABE–Dep. of Chemical Engineering, Faculty of Engineering, University of Porto, Rua Dr. Roberto Frias, Porto, Portugal; 3 Departamento de Informática—Universidade de Vigo, ESEI—Escuela Superior de Ingeniería Informática, Edificio politécnico, Campus Universitario As Lagoas, Ourense, Spain; 4 Centre of Biological Engineering, University of Minho, Campus de Gualtar, Braga, Portugal; Louisiana State University, UNITED STATES

## Abstract

The polymicrobial nature of ventilator-associated pneumonia (VAP) is now evident, with mixed bacterial-fungal biofilms colonizing the VAP endotracheal tube (ETT) surface. The microbial interplay within this infection may contribute for enhanced pathogenesis and exert impact towards antimicrobial therapy. Consequently, the high mortality/morbidity rates associated to VAP and the worldwide increase in antibiotic resistance has promoted the search for novel therapeutic strategies to fight VAP polymicrobial infections. Under this scope, this work aimed to assess the activity of mono- *vs* combinational antimicrobial therapy using one antibiotic (Polymyxin B; PolyB) and one antifungal (Amphotericin B; AmB) agent against polymicrobial biofilms of *Pseudomonas aeruginosa* and *Candida albicans*. The action of isolated antimicrobials was firstly evaluated in single- and polymicrobial cultures, with AmB being more effective against *C*. *albicans* and PolyB against *P*. *aeruginosa*. Mixed planktonic cultures required equal or higher antimicrobial concentrations. In biofilms, only PolyB at relatively high concentrations could reduce *P*. *aeruginosa* in both monospecies and polymicrobial populations, with *C*. *albicans* displaying only punctual disturbances. PolyB and AmB exhibited a synergistic effect against *P*. *aeruginosa* and *C*. *albicans* mixed planktonic cultures, but only high doses (256 mg L^-1^) of PolyB were able to eradicate polymicrobial biofilms, with *P*. *aeruginosa* showing loss of cultivability (but not viability) at 2 h post-treatment, whilst *C*. *albicans* only started to be inhibited after 14 h. In conclusion, combination therapy involving an antibiotic and an antifungal agent holds an attractive therapeutic option to treat severe bacterial-fungal polymicrobial infections. Nevertheless, optimization of antimicrobial doses and further clinical pharmacokinetics/pharmacodynamics and toxicodynamics studies underpinning the optimal use of these drugs are urgently required to improve therapy effectiveness and avoid reinfection.

## Introduction

Ventilator-associated pneumonia (VAP) is a respiratory infectious disease, now recognized as having a polymicrobial nature. VAP occurs 48–72 hours after endotracheal intubation and has an associated estimated mortality of 10–40% [1]. The starting-point for VAP development is the presence of an endotracheal tube (ETT), which allows the leakage of contaminated oropharyngeal secretions down to the lungs and is prone to microbial colonization [2]. A wide spectrum of pathogens is able to attach the ETT surface. *Pseudomonas aeruginosa* stands out in these infections, ranking for higher fatality rates [3], mainly due to its ability to develop biofilms resilient to antibiotic therapy. Isolation of fungal species, such as *Candida albicans*, is also common from tracheal secretions, but usually leads only to the colonization of the airways, rather than causing pneumonia in critically-ill patients [4, 5]. However, the risk of VAP due to infection by *P*. *aeruginosa* is facilitated and markedly increased in patients displaying *C*. *albicans* tracheobronchial colonization [6, 7]. Both *P*. *aeruginosa* and *C*. *albicans* have tendency to form resistant polymicrobial biofilms, playing extensive ecological roles in nosocomial infections, such as VAP [8, 9]. Co-infection by both species has also been well documented, with ample evidence supporting the multifaceted bacterial-fungal and/or bacterial/fungal-host interactions [10–22]. So far, no reliable methods are currently available to detect ETT’s biofilms while the patient remains on invasive mechanical ventilation. Additionally, only few preventive and therapeutic strategies to reduce ETT biofilm formation and VAP have been tested in clinical settings [23–26].

Selecting the appropriate antimicrobial agents and initiating the therapy as early as possible is critical to reduce VAP’s associated mortality [27–29]. Importantly, the choice of the therapy is empirical and dictated by several factors, including: institutional or unit-specific sensitivity testing; patient risk factors; prior cultures or colonization data; duration of the mechanical ventilation; prior exposure to other antimicrobials and severity of the illness. All this information is essential to guide optimal dosage of initial empiric therapy [29, 30]. Although there is no universal regimen for VAP treatment, some recommended therapies stand out [31–33]. Polymyxins are cationic-peptide antibiotics that have re-emerged in later years as the last-resort therapy for respiratory infections caused by multidrug resistant (MDR) Gram-negative bacteria such as *P*. *aeruginosa* [34–36]. The optimization of therapies for *Pseudomonas* species infections is critical due to their prevalent mortality rates comparatively with other pathogens [37, 38].

There is evidence supporting that the initial use of combination drug therapy (i.e. a therapeutic intervention including the administration of more than one drug) can provide a greater spectrum of activity compared with monotherapy in severe infections caused by MDR Gram-negative bacteria [39–44]. Moreover, VAP is associated to biofilms and the persistence of this chronic infection is recurrently attributed to the resilience of polymicrobial biofilms to therapy [45]. The use of antibiotics and antifungals simultaneously or sequentially, for prophylactic and therapeutic purposes, is a common clinical practice in severe infections to face the emergence of resistance to the host immune system response and to antimicrobial therapy [46]. A combination therapy, supported in anti-biofilm antimicrobials together with traditional antibiotics to target cell growth, could be a better alternative to control biofilm-related infectious diseases as VAP. In such combination therapy, the anti-biofilm drugs will impair and/or disturb biofilms contributing to cells leaving the biofilms and entering the planktonic phase, thus removing the additional community level resistance provided by biofilms, and facilitating the targeting of pathogens at the cellular level by traditional antibiotics [47].

Because the emergence and dissemination of antibiotic resistance traits in causal agents in polymicrobial infections and since co-infection by multiple species may result in enhanced pathogenesis and reduce treatment options, it becomes crucial to assess the impact of microorganisms within these polymicrobial communities, by analysing their social interactions, attempting to avoid unsuccessful antibiotherapy and leading to chronic infection suppression.

Based on this, the goal of this study was to exploit the impact of monotherapy *vs* combinational therapy involving an antibiotic agent (Polymyxin B; PolyB) and an antifungal agent (Amphotericin B; AmB) to fight polymicrobial biofilms of *P*. *aeruginosa* and *C*. *albicans*, mixed-communities often retrieved from the VAP’s ETT.

## Material and Methods

### Microorganisms and culture conditions

*P*. *aeruginosa* PAO1 and *C*. *albicans* SC5314, two model reference strains with known sequenced whole genome, were used throughout this work. Both strains were stored at– 80 ± 2°C in broth medium with 20% (v/v) glycerol. Prior to each assay, *P*. *aeruginosa* and *C*. *albicans* strains were subcultured from the frozen stock preparations onto Tryptic Soy Agar (TSA) and Sabouraud Dextrose Agar (SDA) plates, respectively. TSA and SDA were prepared from Tryptic Soy Broth (TSB; Liofilchem, Italy) or Sabouraud Dextrose Broth (SDB; Liofilchem) supplemented with 1.2% w/v agar (Liofilchem). The plates were then incubated aerobically at 37°C for 18–24 h.

Pure liquid cultures (pre-inocula) of *P*. *aeruginosa* were grown overnight in TSB whereas *C*. *albicans* was maintained in SDB. For planktonic and biofilm assays, 0.22 μm filter-sterilized RPMI 1640 medium (Gibco® by Life Technologies ^TM^, Grand Island, NY, USA) at pH 7.0 was used. Unless otherwise stated, all rinse steps were performed either by using 0.9% (w/v) saline solution (NaCl; J.T. Baker, The Netherlands) or ultrapure (UP) sterile water.

### Biofilm formation in vitro

Biofilms were developed according to the modified microtiter plate test proposed by Stepanovic *et al*. [48]. Briefly, both cultures were centrifuged twice (3000 *g*, 4°C, 10 min) and the pellet was ressuspended in RPMI 1640, until reaching 1x10^7^ cells mL^-1^. Bacteria concentration was estimated using an ELISA microtiter plate reader with a wavelength of 640 nm (Sunrise-Basic Tecan, Austria). Yeast cells were enumerated by microscopy using a Neubauer counting chamber. For mixed-species cultures, a combination of 50% of the suspended inoculum of each species was used.

The cellular suspensions were transferred, under aseptic conditions, to 96-well flat tissue culture plates (polystyrene, Orange Scientific, USA) (200 μL per well). To promote biofilm formation, microtiter plates were incubated aerobically for 24 h on a horizontal shaker at 120 rpm and 37°C.

### Biofilm analysis

After biofilm formation, the wells were washed twice with saline solution (200 μL per well) after discarding the planktonic fraction. In order to estimate the number of cultivable biofilm-entrapped cells in single- and mixed-species, the microdrop technique was used. Briefly, 200 μL of fresh saline solution was added to each well and the biofilms were scraped. The scraping technique was previously optimized for *C*. *albicans* and *P*. *aeruginosa* single- and mixed-species biofilms (see S1 Fig in Supporting Information), by measuring the remaining biomass in the microtiter plate wells throughout the crystal violet (CV) staining method, using the procedure previously outlined [49]. In order to ensure the reproducibility of the scraping method, the conditions were strictly followed in all experiments, by using a 200 μL pipette tip and scraping each well for 1 min in the same route and speed. The resulting biofilm-cells suspensions were then serially diluted in saline solution and plated onto non-selective agar (TSB containing agar or TSA for *P*. *aeruginosa* and SDB containing agar or SDA for *C*. albicans pure cultures) plates. Selective agar or *P*. *aeruginosa* (PIA) and *C*. *albicans* (SDA supplemented with 30 mg L^-1^ gentamycin, to suppress the growth of *P*. *aeruginosa*) for colony forming units (CFU) determination was also used. Agar plates were incubated aerobically at 37°C for 24–48 h for cultivable cell counting. Values of cultivable sessile cells were expressed as log_10_ CFU per area (cm^2^).

Stock solutions of two antimicrobial agents, AmB (Sigma-Aldrich, St. Louis, MO, USA) and PolyB (Biochrom GmbH, Berlin, Germany) were prepared, respectively, in dimethyl sulfoxide (DMSO) and in ultrapure distilled water at 5000 mg L^-1^, and stored according to the manufacturer’s instructions.

### Planktonic antimicrobial susceptibilities

The susceptibility of planktonic-cell cultures was evaluated by determining the minimum inhibitory concentration (MIC) and the minimum bactericidal and/or fungicidal concentration (MBC and/or MFC). For simplicity purposes, the abbreviation for minimum microbiocidal concentration (MMC) will be used to refer to MBC and/or MFC. The MIC values were determined according to standard European Committee on Antimicrobial Susceptibility Testing (EUCAST), through the broth microdilution method [50]. Briefly, the initial cell concentration for both microorganisms was adjusted for 1×10^6^ CFU mL^-1^ and dispensed into 96-well plates in a proportion of 1:2 (the final inoculum concentration was 5×10^5^ CFU mL^-1^) with the working antibiotic solutions (previously diluted in RPMI 1640 broth with double of the desired final concentration). Wells containing only broth medium (antibiotic-free medium) worked as negative controls. Plates were incubated overnight at 37°C. MIC was obtained by visual observation of the turbidity gradient. This turbidity shows the capacity of the planktonic cell populations to grow in the presence of the antimicrobials. The minimum concentration where growth inhibition occurs is equivalent to the MIC value.

For the determination of MMC values, 10 μL were removed from each well of the microdilution trays, after incubation, and plated onto TSA (for *P*. *aeruginosa*) and SDA (for *C*. *albicans*) plates and incubated at 37°C. The lowest antimicrobial concentration that yielded no colony growth after 12–24 h was considered as the MMC.

### Antimicrobial therapeutic effect in biofilms

The effect of AmB and PolyB alone was evaluated in single and mixed-species biofilms. For this, 24 h-old biofilms were exposed to increasing concentrations of each antimicrobial agent (1×, 2× and 4× MIC). Specifically, the lowest MIC obtained for each antimicrobial agent in single-species cultures was used (AmB: 0.25 mg L^-1^; PolyB: 2 mg L^-1^). Briefly, after biofilm formation, 100 μL of cell suspension were replaced by the antimicrobial solutions prepared at 2-fold the desired concentration. Plates were then incubated aerobically at 37°C for 48 h. Every 12 h, half of the liquid content of each well was replaced by fresh antimicrobial solution or culture medium (positive control). In addition, every 12 h some biofilms were taken to assess biofilm-cells cultivability through CFU enumeration, as previously described.

### Checkerboard microdilution assay

The combined activity of AmB with PolyB against *P*. *aeruginosa* and *C*. *albicans* mixed-species planktonic cultures was investigated using the standard checkerboard microdilution assays [51, 52]. In brief, serial 2-fold dilutions of AmB and PolyB were mixed together in 96-well microtiter plate such that each row (or column) contained a fixed amount of one agent and increasing amounts of the second agent. The following concentrations range for each antimicrobial agent was tested: 0.0156 to 4 mg L^-1^ for AmB and 0.0156 to 256 mg L^-1^ for PolyB. For each assay, the serial dilutions of each agent were tested individually (to measure the MIC), and control wells containing untreated cells were also grown. Plates were incubated for 24 h at 37°C under static conditions, and the MIC of each antimicrobial agent alone was determined as well as the MIC of the agents in combination. The fractional inhibitory concentration (FIC) was calculated for each well along with the growth–no growth interface (corresponding to ~ 90% inhibition in the presence of the combination, with each agent below its own individual MIC). For agents A and B, the FIC of the combination is calculated as previously [44, 53, 54]:

$$\sum FIC_{A+B}=FIC_{A}+FIC_{B}$$

where FIC_A_ = MIC_A_ combined/MIC_A_ alone and FIC_B_ = MIC_B_ combined/MIC_B_ alone. For interpretation purposes, the ΣFIC ⩽ 0.5 indicates a synergistic effect; between 0.5 and 1 is assumed to be an additive effect; between 1 and 4 means indifference; and greater than 4 symbolizes antagonism effect among both drugs [55].

### Combinatorial effect of antimicrobial agents on biofilms

Based on the FIC index results, the combinatorial effect of AmB and PolyB (0.016 mg L^-1^ AmB + 8 mg L^-1^ PolyB; 0.016 mg L^-1^ AmB + 32 mg L^-1^ PolyB; and 0.016 mg L^-1^ AmB + 256 mg L^-1^ PolyB) was assessed against 24 h-old dual-species biofilms following a procedure similar to the individual application of the antimicrobials.

### Time-kill kinetics

To examine the rate of killing in mixed-species biofilm populations of the AmB and PolyB synergistic combination, time-kill assays were performed. Briefly, 96-well microtiter plates containing the preformed biofilms of *P*. *aeruginosa* and *C*. *albicans* were challenged using synergistic combinations (from the checkerboard assays), with concentrations of the individual agents alone, and untreated cells as controls. Plates were then incubated at 37°C for 24 h under static conditions and CFU cm^-2^ were estimated for each species (by using selective agar media) at 2 h-time points up to 24 h.

### Fluorescence in situ hybridization (FISH) applied to biofilms

In order to discriminate among bacterial and fungal populations within the polymicrobial biofilms, FISH was employed using a red-fluorescent labelled peptide-nucleic acid (PNA) probe to specifically detect *P*. *aerugionosa*. This PNA probe, designated as Paer565, was previously designed, optimized and validated on biofilms by Lopes et al. [56]. Briefly, dual-species biofilms of *P*. *aeruginosa* and *C*. *albicans* were formed in polystyrene (PS) coupons (1×1 cm) placed in the bottom of the wells of 24-well microtiter plates. The fungal population was identified by counterstaining the samples with 4`, 6-diamidino-2-phenylindole (DAPI; Sigma, St. Louis, MO, USA) blue staining at the end of the hybridization procedure. After biofilm formation, the PS surfaces were washed twice with 1 mL sterile distilled water and allowed to dry (~60°C) for 15 min. The biofilm was fixed with methanol (100% v/v) for 20 min. This initial step of fixing the biofilm with methanol is essential to avoid the detachment of cells during the hybridization procedure. Afterwards, 30 μL of each solution of 4% (w/v) paraformaldehyde followed by 50% (v/v) ethanol was dispensed in the PS coupons for 10 min each and allowed to air dry. Subsequently, 20 μl of hybridization solution containing the PNA probe at 200 nM were dispensed on the coupons, which were finally covered with coverslips and incubated in the dark for 1h at 65°C. Soon after hybridization, PS coupons were carefully removed and were immersed for 30 min in 24-well plates containing 1 mL per well of a prewarmed (65°C) washing solution composed of 5 mM Tris Base, 15 mM NaCl and 1% (vol/vol) Triton X-100 (all from Sigma- Aldrich, St. Louis, MO, USA). The PS coupons were removed from the plates and allowed to air dry in the dark before counterstaining with DAPI. For this, each coupon was covered with 20 μL of DAPI (40 μg mL^-1^) for 5 min at room temperature in the dark before immediate observation in the fluorescence microscope. Negative controls were assessed for each experiment, without probe added to the hybridization solution. For microscopic visualization, a fluorescence microscope (Olympus BX51, Perafita, Portugal), equipped with the filters sensitive to DAPI (BP 365–370, FT 400, LP 421) and to the signalling molecule of the red-fluorescent PNA probe (BP 530–550, FT 570, LP 591, for Alexa Fluor 594).

### Cell viability assessment of biofilm-embedded cells

In order to evaluate the cell viability of polymicrobial biofilms after treatment, the Live/Dead® BacLight™ Bacterial Viability Kit (Molecular Probes, Leiden, Netherlands) was employed. Basically, biofilms were formed on PS coupons, as described above, and were then stained for 15 min in the dark with a mixture of the SYTO 9 and Propidium Iodide, both prepared at 3μL mL^-1^ in saline solution. For microscopic observation, an Olympus BX51 microscope fitted with fluorescence illumination was used. The optical filter combination consisted of 470 to 490 nm in combination with 530 to 550 nm excitation filters.

### Statistical analysis

Data were analyzed using the Prism software package (GraphPad Software version 6.0 for Macintosh). One-way ANOVA tests were performed and means were compared by applying Tukey`s multiple comparison test. The statistical analyses performed were considered significant when *P*<0.05. For all assays, at least three independent experiments were carried out.

## Results

### Susceptibility testing of planktonic populations

The MIC and MMC of AmB and PolyB against planktonic populations of *P*. *aeruginosa* and *C*. *albicans* are summarized in Table 1.

**Table 1:** Susceptibility profiles of single- and dual-species planktonic cultures of *P*. *aeruginosa* and *C*. *albicans* towards AmB and PolyB. MIC and MMC are expressed in mg L^-1^.

		Single-species cultures		Dual-species cultures		
		*P*. *aeruginosa*	*C*. *albicans*	*P*. *aeruginosa*		*C*. *albicans*
**AmB**						
	**MIC**	≥ 16	0.25	≥ 16		
	**MMC**	≥ 16	0.25	≥ 16	2	
**PolyB**						
	**MIC**	2	256	512		
	**MMC**	4	256	4	512	

As expected, AmB was more effective against *C*. *albicans* (MIC: 0.25 mg L^-1^), and PolyB against *P*. *aeruginosa* (MIC: 2 mg L^-1^). For mixed cultures, both agents required equal or even higher doses than those required to inhibit the single populations. The MMC values were similar to MIC for single-species populations. Similarly, the MMC of *P*. *aeruginosa* in mixed culture was not altered in comparison with single populations, whereas an increase in the MMC (2-fold for PolyB and 8-fold for AmB) was observed for *C*. *albicans*.

### Therapeutic effect of antimicrobials in single- and dual-species biofilms

**Fig 1:**
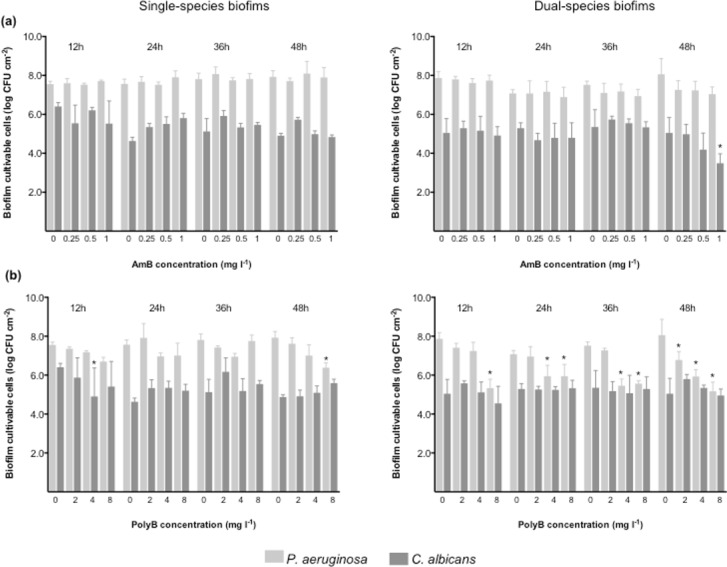
Therapeutic effect of AmB (a) and PolyB (b) against 24 h-old single- and dual-species biofilms formed by *P*. *aeruginosa* and *C*. *albicans*. *P<0.05 indicates a statistically different reduction in comparison with the respective control (corresponding to 0 mg L^-1^).

The therapeutic effect of AmB and PolyB at increasing concentrations was followed for 48 h on 24-h-old mono- and dual-species biofilms of *P*. *aeruginosa* and *C*. *albicans* by determining biofilm CFU numbers at each 12 h (Fig 1).

As can be observed, for each time point, the CFU number obtained for *P*. *aeruginosa* and *C*. *albicans* in single- and in mixed-species biofilms was not significantly disturbed (Fig 1). In general, neither AmB nor PolyB had a time-dependent therapeutic effect against single- and mixed-species biofilms. No significant perturbations were observed in *C*. *albicans* biofilm cell numbers, with both antimicrobial agents causing only punctual reductions in this species. Similar results were observed for AmB in *P*. *aeruginosa* biofilms. However, PolyB promoted significant and gradual reductions in *P*. *aeruginosa*, with the highest concentrations (4 and 8 mg L^-1^) leading to major cell reductions (≥ 2 log) (P<0.05), particularly for mixed-species biofilms.

### Evaluation of the antimicrobials potential synergy

The combination effect of AmB with PolyB on planktonic growth of dual-species cultures involving *P*. *aeruginosa* and *C*. *albicans* was investigated using the checkerboard microdilution assay. Table 2 summarizes the MIC values that were obtained for each antimicrobial agent when combined, and that resulted in the lowest ΣFIC and best outcome against dual-species planktonic cultures.

**Table 2:** Values of MIC obtained for the combinational activities of AmB and PolyB against dual-species planktonic cultures formed by *P*. *aeruginosa* and *C*. *albicans*. The lowest values of the sum of the fractional inhibitory concentration indexes, ΣFIC, for each antimicrobial agent and the best outcome are shown.

	**MIC (mg L**^**-1**^**)**	**ΣFIC (mg L**^**-1**^**)**	**Outcome**
**AmB/PolyB combination**	0.0156/1	0.066	Synergistic

The combination of AmB with PolyB resulted in a synergistic outcome against the mixed-species planktonic cultures, reaching a ΣFIC below 0.5 (ΣFIC: 0.066 mg L^-1^). Thus, MICs were significantly reduced when AmB and PolyB were combined (AmB: from ≥16 to 0.0156 mg L^-1^ for AmB and from 512 to 1 mg L^-1^ for PolyB for PolyB, for a single and combined application, respectively). These results indicate enhanced effectiveness of AmB/PolyB combination against planktonic cells of *P*. *aeruginosa*+*C*. *albicans* cultures.

### Effect of antimicrobial combination activity against dual-species biofilms

**Fig 2:**
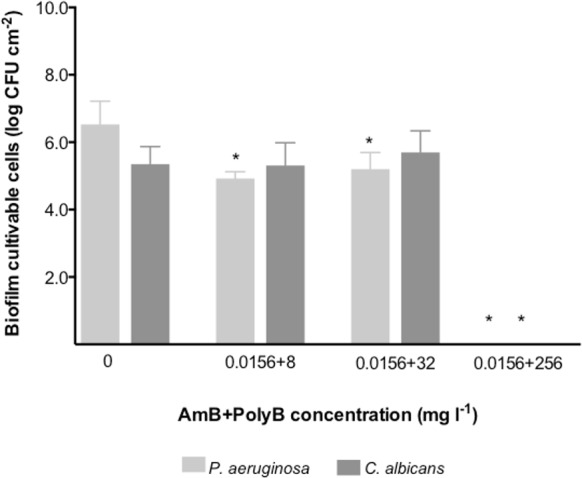
Effect of AmB and PolyB, combined at different concentrations, against 24 h-old dual-species biofilms formed by *P*. *aeruginosa* and *C*. *albicans*. *P<0.05 indicates statistically different reduction in comparison with the respective control (corresponding to 0 mg L^-1^).

Because AmB/PolyB combination showed synergistic activity against mixed-species planktonic cultures, the efficacy of the antifungal-antibacterial combination was further inspected in preformed biofilms of *P*. *aeruginosa* and *C*. *albicans* (Fig 2).

Taking into account the antimicrobials potential synergy results, it was interesting to inspect the effect of AmB/PolyB combination in preformed mixed-species biofilms of *P*. *aeruginosa* and *C*. *albicans*. Due to the high risk of dose-dependent AmB nephrotoxicity [57, 58], we combined AmB at a fixed concentration (0.0156 mg L^-1^) with increasing doses of PolyB (8, 32 and 256 mg L^-1^). The more interesting outcome was observed with the combination of 0.0156 mg l^-1^ of AmB with the highest PolyB concentration tested (256 mg l^-1^), promoting the eradication of the number of *C*. *albicans* and *P*. *aeruginosa* cells entrapped in mixed-species biofilms in comparison with the respective control biofilms.

**Fig 3:**
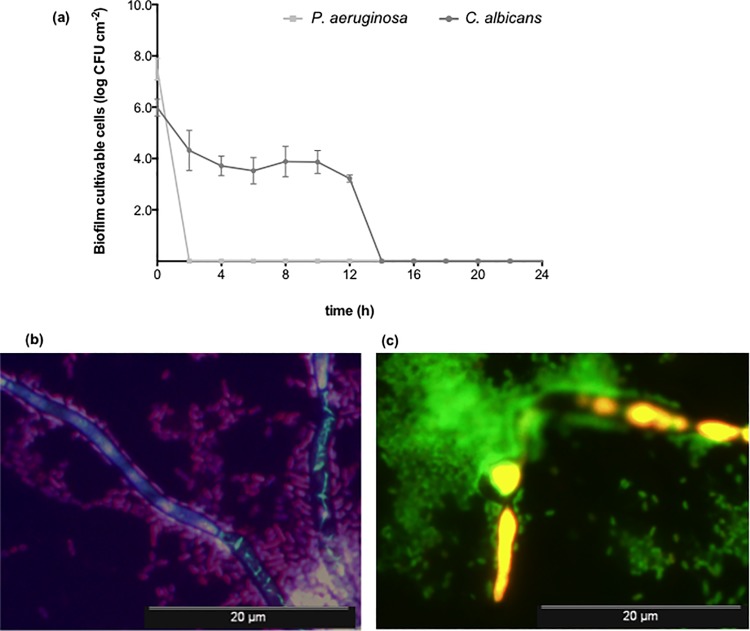
Time-kill kinetics obtained for the combinatorial activity of AmB (at 0.0156 mg L^-l^) and PolyB (at 256 mg L^-l^) against dual-species biofilms of *P*. *aeruginosa* and *C*. *albicans* (a) and epifluorescence images from mixed *C*. *albicans* and *P*. *aeruginosa* biofilms 24 h post-treatment discriminated by PNA FISH assay (b) and stained with LIVE/DEAD® staining system (c).

Since total inhibition of the mixed-species consortia was accomplished with the combinatorial activity of 0.0156 mg l^-1^ AmB and 256 mg l^-1^ PolyB, the time-kill kinetics (Fig 3) was assessed for this formulation against the mixed-species consortia. The rationale was to investigate the time point where the inhibition of both species occurred. Fig 3A demonstrates an almost immediate elimination of *P*. *aeruginosa* cells after only 2 h of treatment. Conversely, for *C*. *albicans*, a CFU reduction occurred gradually until 14 h. To discriminate both *P*. *aeruginosa* and *C*. *albicans* species in these treated biofilms, and to evaluate the biofilm-cell viability, fluorescent *in situ* hybridization (FISH) using a *P*. *aeruginosa* PNA probe and the LiveDead *Bac*Light Bacterial Viability Kit were respectively employed. After 24 h of treatment with the AmB/PolyB combination, both species could be discriminated (Fig 3B) with a large number of *P*. *aeruginosa* cells (detected by a PNA red-labelled PNA probe) being preferably located around the *C*. *albicans* hyphae (detected by blue DAPI staining). Additionally, the LiveDead staining method indicated that the green bacterial cells were more abundant comparatively to *C*. *albicans*, meaning that they were viable even after 24 h of treatment with AmB+PolyB combination (Fig 3C). This seems to indicate that, even though *P*. *aeruginosa* lost its culture capability immediately 2 h post-treatment, it remained in a viable state.

## Discussion

The growing evidence that most respiratory infections are developed throughout complex processes involving several pathogens [59] could partially explain the lack of response to conventional therapeutic regimens that primarily target single causative agents instead of all members in the consortia. So, it is strongly suggested that the antimicrobial therapy outcome in mixed-species infections may be severely impacted by the number, type and interplay of microorganisms within the polymicrobial communities. In light of the recent reports regarding complex interactions between *P*. *aeruginosa* and *C*. *albicans* in VAP [60–62], the present study intended to understand how these species respond to individual and combined antimicrobial therapy using antibiotic and antifungal agents.

According to the epidemiological breakpoints set by EUCAST [63, 64] results obtained from this study demonstrated that *C*. *albicans* planktonic cultures were susceptible to AmB, whereas *P*. *aeruginosa* presented sensitivity to PolyB (Table 1). As expected, the antifungal agent presented reduced activity against *P*. *aeruginosa* (MIC and MMC ≥ 16 mg l^-1^), whilst the antibacterial agent did not promote an effect on *C*. *albicans*, showing high MIC and MMC values (256 mg l^-1^). AmB, a polyene antifungal, has been reported as the gold standard in cases of serious and invasive *Candida* infections, due to its remarkably low level of resistance amongst fungal species and its fungicidal mechanisms that account for broad-spectrum coverage [65]. AmB exerts fungicidal activity by inducing the formation of pores on the fungal cell membrane due to interaction with membrane-bound ergosterol and subsequent loss of cytoplasmatic content and cellular loss of viability [66]. Bacteria are not affected, as their cell membranes do not contain sterols. In turn, PolyB is a cationic antimicrobial peptide that is now widely used as a therapeutic agent against Gram-negative infections. It generally causes disruption of the cytoplasmic membrane of bacteria, but can also inhibit intracellular processes such as nucleic acid, DNA, or protein synthesis [67]. In this study, the lower efficiency of PolyB in eukaryotes could be partly due to the presence of sterols in the eukaryotic membrane, as sterols have been shown to reduce the insertion of cationic peptides into anionic membranes to form pores [68–72]. Besides that, the antifungal properties of PolyB have now become widely explored [73–78]. But the potential of PolyB as an antifungal agent alone generally demands high doses to inhibit such infections and combined therapy of PolyB with other agents (e.g. azoles) will probably have a far-reaching impact on the development of novel, more effective and safer antifungal therapies [76, 79]. In this study, higher concentrations of antimicrobials were required to inhibit mixed-species populations in comparison with the mono-microbial counterparts (Table 1). Specifically, 2- and 8-fold increases were observed in the MMC for PolyB and AmB, respectively, against *C*. *albicans*. A recent study has already shown a similar increase in the AmB MMC against *C*. *albicans* mixed planktonic populations [80]. This increase on antimicrobial tolerance observed in mixed cultures can be related to species-related resistance mechanisms (e.g. the difficulty of the interactions between the antimicrobial agents and target sites; the efflux of the antimicrobial agent from the bacterial cells before reaching target sites; and/or the destruction or modification of the antimicrobial molecule), in addition to resistance factors provided by microorganisms when in mixed cultures (e.g. protection by one of the species; cell rearrangement; interactions among the resident species; among others). In fact, microbes involved in polymicrobial infections may often display interactions that can alter the course of pathogenesis in polymicrobial communities and exert effects on microbial behaviour, dissemination, survival, the response to antimicrobials and, ultimately, patient prognosis [81]. Still, the molecular mechanisms governing these interactions are not well understood.

Interactions between prokaryotes and eukaryotes are ubiquitous in nature and are important for the survival of species and ecological balance. When in biofilms, the situation becomes more complex since a range of metabolic interactions among the resident species may occur, influencing the behaviour of the whole community [82, 83]. In this study, the simultaneously presence of both bacterial and fungal species did not result in significant changes in the overall consortia (Fig 1), with the CFUs estimated for each species in single and mixed untreated biofilms not significantly changed over time. This result strongly suggests that any species had interference in the growth of the other within the co-cultures. Concerning the antimicrobial application, AmB and PolyB alone were not enough to eradicate *P*. *aeruginosa* and *C*. *albicans* biofilms (Fig 1). *C*. *albicans* was persistent to the action of both antimicrobial agents, with only AmB showing merely punctual and less significant CFU reductions. Previous studies have shown that AmB generally requires high concentrations, even above the therapeutic range, to initiate a therapeutic effect in *C*. *albicans* biofilms [84, 85]. In contrast, PolyB could trigger significant disturbances on *P*. *aeruginosa*, namely when growing under mixed biofilms. Resistance is reportedly up to 10–1000 fold greater in biofilms when compared with planktonic cultures, which could be the explanation for the frequent therapeutic failure of antimicrobials against biofilm infections [86–91] and specifically biofilm cells of *P*. *aeruginosa* and *C*. *albicans*, frequently colonizing the VAP ETT, can be significantly more resistant or tolerant towards antimicrobial agents [92–95]. Overall, an antimicrobial delayed penetration within the biofilm and slower growth rates within the depths of the biofilm due to depletion of organic nutrients, inorganic ions, and oxygen are some of the resistance factors conventionally associated to biofilms [96–98].

Since *C*. *albicans* often develops polymycrobial biofilms with *P*. *aeruginosa* in the VAP ETT, and knowing that AmB and PolyB alone showed low efficiency against these consortia, it was relevant to test multidrug treatment strategies, by combining both antimicrobial agents to fight these mixed infections and impair resistance evolution. Combination therapy may result in a synergistic effect of the drugs, and at the same time prevent the emergence of resistance during therapy. Another reason to use combination therapy is to provide a broad-spectrum empiric regimen that is likely to include at least one drug that is active against the MDR etiologic agent [29]. In this study, AmB and PolyB exhibited an *in vitro* synergistic effect against *C*. *albicans* and *P*. *aeruginosa* planktonic mixed cultures (Table 2). When applied in polymicrobial biofilms, the combination using the highest concentration of PolyB (256 mg l^-1^) could eradicate the whole biofilm (Fig 2). It is important to highlight, however, that the effective dose of PolyB was higher than the one allowed for clinical use, likely causing strong toxicity for humans [99, 100], and there is thus urgency to optimize the clinical use of PolyB by designing effective combination therapies. Moreover, an equitative inoculum proportion of each species was used in this study and might not represent the real species proportion in VAP infection. In a real clinical scenario, where a co-colonization (with a sequential inoculum of each species involved), the efficacy of AmB and PolyB combination treatment should be confirmed, in order to understand the mechanisms involved in making *P*. *aeruginosa-C*. *albicans* co-exist successfully in infection.

Since the total inhibition of *P*. *aeruginosa* and *C*. *albicans* polymicrobial consortia was obtained with the combined activity of 0.0156 mg l^-1^ AmB and 256 mg l^-1^ PolyB (Fig 2), it was further investigated at which time point it was achieved by performing time-kill kinetics (Fig 3A). Whilst *P*. *aeruginosa* cells were inhibited 2 h post-treatment, *C*. *albicans* were only eliminated 14 h after treatment. The application of PNA-FISH and LiveDead assays after 24 h-treatment demonstrated that both microbial species were present in the consortium, and bacterial cells within polymicrobial biofilms were still viable even after treatment. Therefore, these results indicate that those cells were undergoing a viable but not cultivable (VBNC) state. It is important to note that the viability kit used in this assay is specific for bacterial species, hence the colour of the hyphae cells in the image could not match the reality of the *C*. *albicans* cell state in polymicrobial biofilms. The VBNC state is a unique survival strategy adopted by many bacteria, including *P*. *aeruginosa*, in response to adverse environmental conditions such as antimicrobial pressure, high/low temperature, starvation, chlorination, change in the pH, and oxygen stress [101–105]. Yeasts are also capable to undergo a VBNC state throughout the same reasons [106–108]. Additionally, the ability of microorganisms to enter the VBNC state may be advantageous for cells, but the underestimation or non-detection of viable cells in clinical samples induces a serious risk to human health. The risks appear from the fact that the pathogenic microorganism can be virulent in the VBNC state or recover virulence after re-acquired cultivability under suitable conditions. Moreover, the inherent characteristics of VBNC cells may lead to latency and consequently to worsening disease even in patients already subjected to antimicrobial treatment.

Also, fluorescence images (performed *in situ*) allowed observing the distribution of the different populations within the mixed-species biofilm, with *P*. *aeruginosa* surrounding and colonizing *C*. *albicans* hyphae. This is a common phenomenon already described in other studies [18, 109], which is frequently mediated by quorum sensing molecules [19]. It is important to highlight that the ability of *C*. *albicans* to switch from the planktonic single yeast cell to hyphal morphologies has a major influence on its virulence [21, 110–113]. It is suggested that this morphological plasticity may even confer aggressiveness to *C*. *albicans* colonization and reflect tolerance to treatment. Here, it was observed that the *C*. *albicans* strain used in this study could produce hyphae under unstressed and stressed situations, as previously demonstrated [114] which could contribute to its high resilience towards antimicrobial treatment and posterior consecutive recoveries of its regrowth aptitude.

## Conclusions

The role of polymicrobial biofilms in infectious diseases, such as VAP, is of utmost importance and will probably direct novel therapies that target the multiplicity of species within the consortia by avoiding the enhanced pathogenesis that results from interactions among the causative microbes of such infections. The increased incidence of drug resistant fungi and bacteria in polymicrobial consortia has resulted, in part, from the intense use of antifungals and antibiotics in clinical settings. For therapies to be maximally effective, however, anti-biofilm therapeutic interventions will be required. Here, combination of an antibiotic agent (PolyB) and an antifungal agent (AmB) had shown a potential synergy therapeutic effect against polymicrobial communities involving *P*. *aeruginosa* and *C*. *albicans*. However, for clinical application purposes, such combination should be optimized (e.g. antimicrobial concentrations, and timing of administration) to avoid reinfection.

Although the combination therapy is not used in clinical practice given the associated limitations, this has shown a high potential for treatment of VAP in the future. As such, the optimization of this therapy and research of new anti-biofilm effective antimicrobials, as well as new anti-virulence drugs, are required to ensure successful treatment of these polymicrobial infections.

## Supporting Information

S1 Fig Effect of the scraping method in biofilms. **(A)** Quantification and **(B)** visualization of *P*. *aeruginosa* (PA) and *C*. *albicans* (CA) biomass in single- and mixed-species biofilms remaining in the microtiter plate wells after the scraping method. The remaining biomass adhered to the microtiter plate wells was quantified by using the crystal violet (CV) staining method and compared with the biomass in non-scraped wells. In (B), the left columns represent controls (no-scraping), whereas the right ones represent the remaining biomass after scraping for each biofilm. The column indicated by the symbol (-) is for the negative control (only culture medium).
(PDF)
