# Supplementary figures and images for: Polymicrobial Ventilator-Associated Pneumonia: Fighting In Vitro Candida albicans-Pseudomonas aeruginosa Biofilms with Antifungal-Antibacterial Combination Therapy
Source: PLoS One. 2017 Jan 23;12(1):e0170433. doi: 10.1371/journal.pone.0170433 (PMC5256963; doi:10.1371/journal.pone.0170433)

(A)

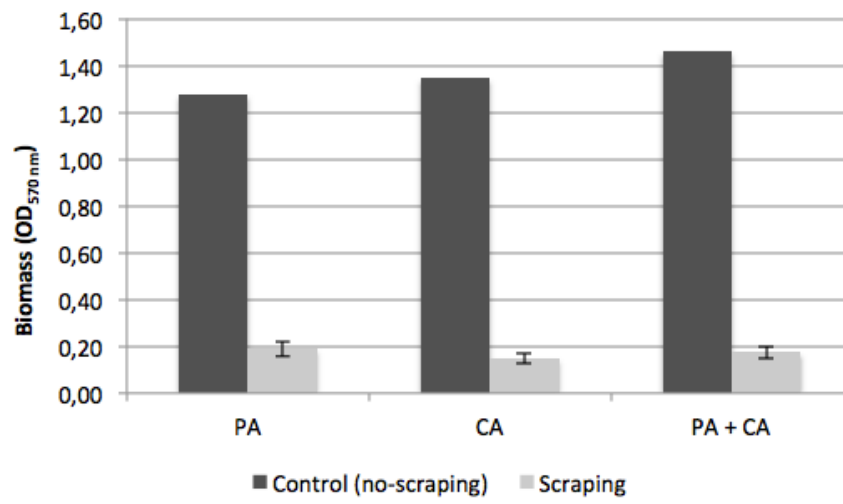

(B)

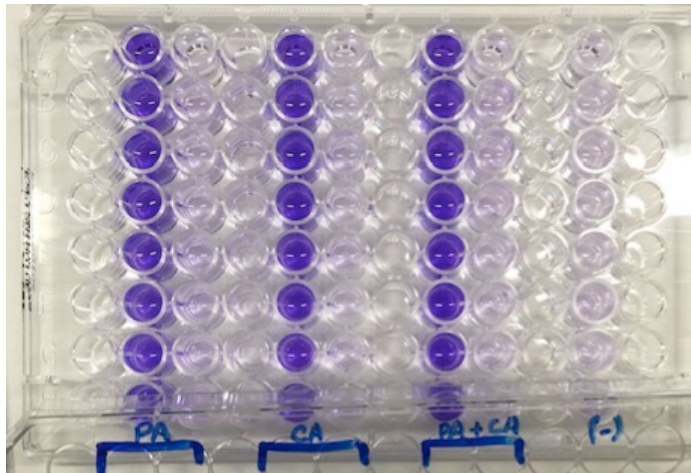

Supplement: S1 Fig — (A) Quantification and (B) visualization of P. aeruginosa (PA) and C. albicans (CA) biomass in single- and mixed-species biofilms remaining in the microtiter plate wells after the scraping method. The remaining biomass adhered to the microtiter plate wells was quantified by using the crystal violet (CV) staining method and compared with the biomass in non-scraped wells. In (B), the left columns represent controls (no-scraping), whereas the right ones represent the remaining biomass after scraping for each biofilm. The column indicated by the symbol (-) is for the negative control (only culture medium). (PDF) [file pone.0170433.s001.pdf]
